# Impairment of cerebellar long-term depression and GABAergic transmission in prion protein deficient mice ectopically expressing PrPLP/Dpl

**DOI:** 10.1038/s41598-020-72753-6

**Published:** 2020-09-28

**Authors:** Yasushi Kishimoto, Moritoshi Hirono, Ryuichiro Atarashi, Suehiro Sakaguchi, Tohru Yoshioka, Shigeru Katamine, Yutaka Kirino

**Affiliations:** 1 Laboratory of Neurobiophysics, Kagawa School of Pharmaceutical Sciences, Tokushima Bunri University, Sanuki, Kagawa 769-2193 Japan; 2 Department of Physiology, Faculty of Medicine, Wakayama Medical University School of Medicine, Wakayama, 641-8509 Japan; 3 Division of Microbiology, Department of Infectious Diseases, Faculty of Medicine, University of Miyazaki, Miyazaki, 889-2192 Japan; 4 Division of Molecular Neurobiology, Institute for Enzyme Research (KOSOKEN), Tokushima University, Tokushima, 770-8501 Japan; 5 Center of Excellence for Environmental Medicine, Kaohsiung Medical University, Kaohsiung, 807 Taiwan; 6 Center for International Collaborative Research, Nagasaki University, Nagasaki, 852-8523 Japan

**Keywords:** Cognitive ageing, Neural circuits, Synaptic transmission, Synaptic plasticity, Long-term depression, Neuroscience, Classical conditioning

## Abstract

Prion protein (PrP^C^) knockout mice, named as the “Ngsk” strain (Ngsk *Prnp*^0/0^ mice), show late-onset cerebellar Purkinje cell (PC) degeneration because of ectopic overexpression of PrP^C^-like protein (PrPLP/Dpl). Our previous study indicated that the mutant mice also exhibited alterations in cerebellum-dependent delay eyeblink conditioning, even at a young age (16 weeks of age) when neurological changes had not occurred. Thus, this electrophysiological study was designed to examine the synaptic function of the cerebellar cortex in juvenile Ngsk *Prnp*^0/0^ mice. We showed that Ngsk *Prnp*^0/0^ mice exhibited normal paired-pulse facilitation but impaired long-term depression of excitatory synaptic transmission at synapses between parallel fibres and PCs. GABA_A_-mediated inhibitory postsynaptic currents recorded from PCs were also weakened in Ngsk *Prnp*^0/0^ mice. Furthermore, we confirmed that Ngsk *Prnp*^0/0^ mice (7–8-week-old) exhibited abnormalities in delay eyeblink conditioning. Our findings suggest that these alterations in both excitatory and inhibitory synaptic transmission to PCs caused deficits in delay eyeblink conditioning of Ngsk *Prnp*^0/0^ mice. Therefore, the Ngsk *Prnp*^0/0^ mouse model can contribute to study underlying mechanisms for impairments of synaptic transmission and neural plasticity, and cognitive deficits in the central nervous system.

## Introduction

Over the past few decades, various independent lines of mice lacking prion protein (PrP^C^) have been generated to evaluate the role of this protein^1–3^. Most lines of PrP^C^ KO mouse show neuronal dysfunction, such as impaired long-term potentiation, and motor incoordination, and altered circadian rhythm^4^. Among the PrP^C^ KO mouse lines, the locus *Prnd*, which is 16 kb downstream of *Prnp* and encodes the 179 residue PrP-like protein Doppel (PrPLP/Dpl), were ectopically expressed in the brain of Ngsk *Prnp*^0/0^ mice^2,5^, but not in the brain of ZrchI *Prnp*^0/0^ mice. Ngsk *Prnp*^0/0^ mice exhibited drastic neuronal changes of late-onset cerebellar Purkinje cell (PC) degeneration^6^, possibly because of both the functional loss of PrP^C^ and/or overexpression of PrPLP/Dpl in the cerebellum^7,8^. Indeed, PrP^C^ is thought to have neuroprotective properties against oxidative stress^9^.

Our previous study demonstrated that the Ngsk *Prnp*^0/0^ mice exhibited age-dependent alterations in cerebellum-dependent eyeblink conditioning in 2 indices: the conditioned response (CR) probability and timing of CR expression^10^. Ngsk *Prnp*^0/0^ mice at the age of 16 weeks exhibited apparently faster CR acquisition but a lower CR amplitude and impaired adaptive CR timing^10^.

PrP^C^ is highly expressed in normal cerebellar Purkinje cells (PCs) and granule cells^11,12^, indicating that the protein plays a role in normal cerebellar synaptic function and neuronal plasticity. Although electrophysiological studies of cerebellar function have been already performed in ZrchI *Prnp*^0/0^ mice, the physiological properties of cerebellar PCs in Ngsk *Prnp*^0/0^ mice have not been characterized^13–20^. Therefore, in the present study, we examined whether a deficiency in PrP^C^ and ectopic expression of PrPLP/Dpl in Ngsk *Prnp*^0/0^ mice affect cerebellar physiological functions by evaluating the basic excitatory and inhibitory synaptic transmission to PCs and long-term depression (LTD) of excitatory synaptic transmission at parallel fibre (PF)-PC synapses. We found that Ngsk *Prnp*^0/0^ mice showed not only weakened GABA_A_-mediated inhibitory postsynaptic currents in PCs but also impaired LTD, suggesting that PrPLP/Dpl expression can induce cerebellar dysfunctions by impairing cerebellar synaptic transmission.

## Results

### Normal synaptic transmission and altered IPSCs in Ngsk Prnp^0/0^ PCs

**Figure 1:**
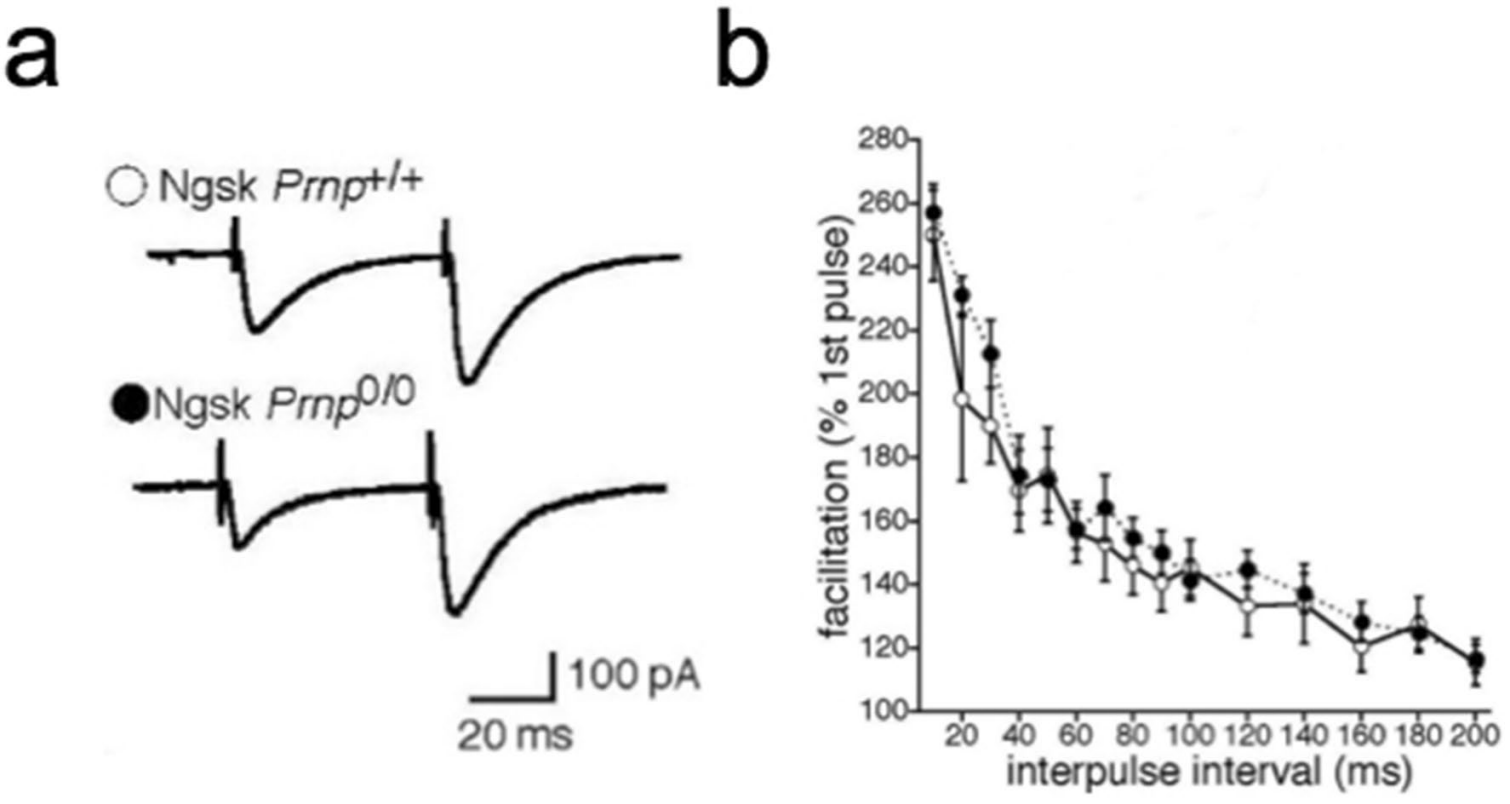
Normal paired pulse facilitation of PF-EPSCs in PCs of Ngsk *Prnp*^0/0^ mice. (**a**) Representative traces of PF-EPSCs induced by paired-pulse stimulation in PCs from control (upper) and Ngsk *Prnp*^0/0^ (lower) mice. (**b**) Paired pulse facilitation (PPF) of PF-EPSCs (expressed as the ratio of the responses to the first and second pulses) in PCs from the control (open circle, *n* = 13 from 9 mice) and Ngsk *Prnp*^0/0^ (closed circle, *n* = 12 from 7 mice) mice is plotted as a function of the interpulse interval. Results indicate the mean ± SEM.

**Figure 2:**
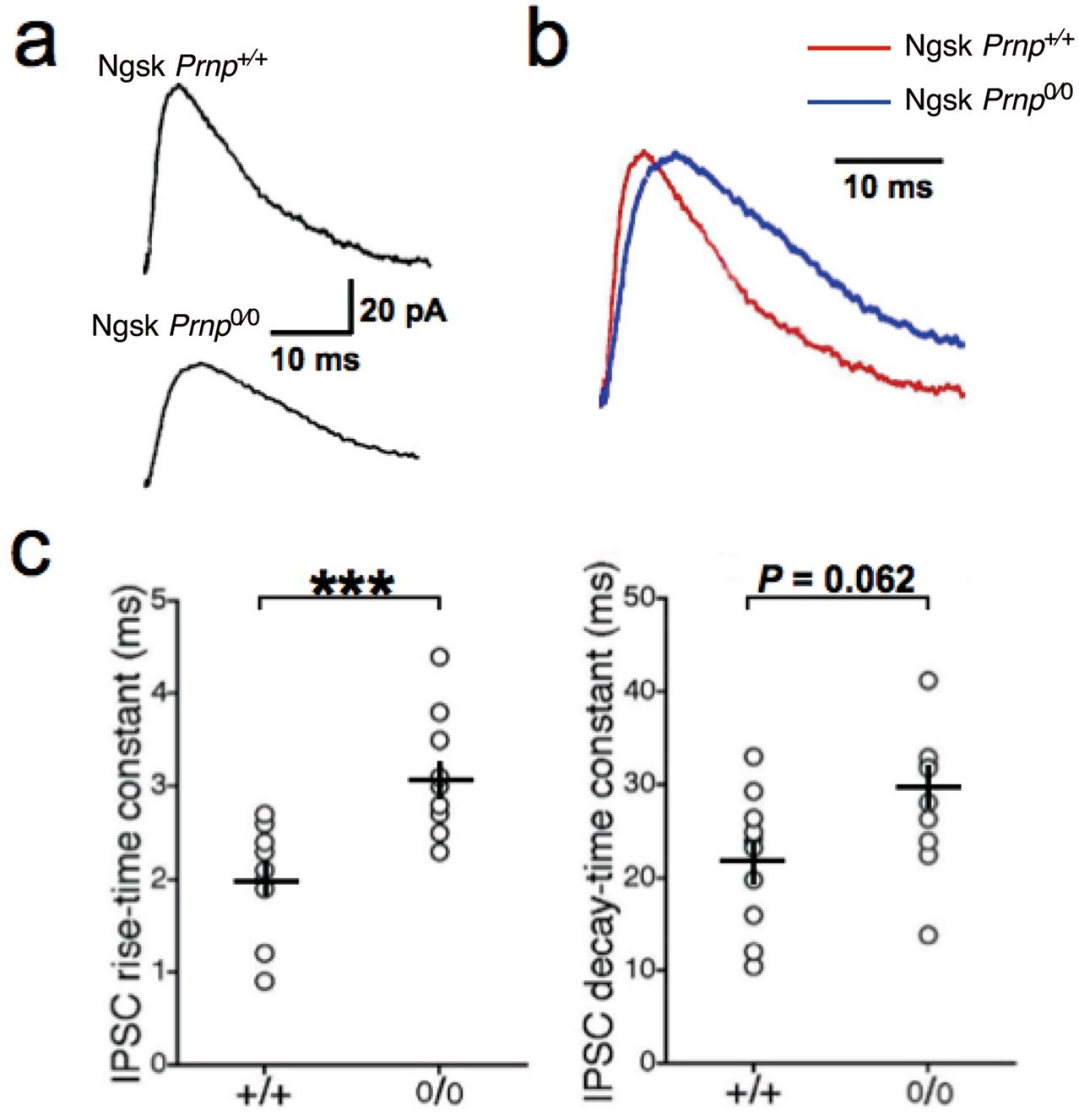
Altered kinetics of parameters of IPSCs in PCs of Ngsk *Prnp*^0/0^ mice. Pharmacologically isolated monosynaptic IPSCs were recorded from PCs voltage-clamped at – 50 mV of the control (*n* = 10 from 6 mice) and Ngsk *Prnp*^0/0^ mice (*n* = 10 from 5 mice). (**a**) The averaged traces of 10 consecutive IPSCs for control slices (upper) and Ngsk *Prnp*^0/0^ slices (lower) were shown as the outward current. (**b**) The averaged traces in (**a**) are scaled and superimposed to compare their shapes. IPSCs from Ngsk *Prnp*^0/0^ mice (blue) were slower than those from control mice (red). (**c**) The rise-phase time constant (left) was slower in Ngsk *Prnp*^0/0^ mice, while the decay-phase time constant (right) did not differ between the two groups. Data points represent individual cells, with the black line representing the mean ± SEM. ***P < 0.001.

First, to examine synaptic function at PF-PC synapses in Ngsk *Prnp*^0/0^ mice, we measured PF-excitatory postsynaptic currents (EPSCs) and obtained their rise and decay time constants and paired-pulse facilitation (PPF). It is noted that PF-induced EPSCs are measured in the presence of bicuculline (10 μM) in ACSF to abolish IPSCs. The mean rise time constant of EPSCs, calculated using a single exponential fit, was 2.04 ± 0.37 ms (n = 13) and 1.69 ± 0.33 ms (*n* = 12) in cerebellar PCs from Ngsk *Prnp*^+/+^ (control) and Ngsk *Prnp*^0/0^ mice, respectively. The mean decay time constant was 15.5 ± 1.1 ms (*n* = 13) in the control slices vs. 18.4 ± 2.1 ms (*n* = 12) in the Ngsk *Prnp*^0/0^ slices. There was no significant difference in either the rise or decay time constants between cerebellar PCs from control and Ngsk *Prnp*^0/0^ slices (*p* = 0.74, 0.52, respectively). The PF-evoked responses exhibited PPF, which is considered to arise from increased transmitter release from PF terminals. The PPF decreased with interpulse intervals in a similar manner as in control and Ngsk *Prnp*^0/0^ mice (*p* > 0.05, Fig. 1a,b). Thus, the short-term synaptic plasticity of PF-PC synapses appeared to be normal in Ngsk *Prnp*^0/0^ mice. Furthermore, no significant difference was found in the resting membrane potentials (– 51.5 ± 1.1 mV, n = 13 for control slices vs – 55.4 ± 1.5 mV, n = 12 for Ngsk *Prnp*^0/0^ slices *p* = 0.62). However, monosynaptic GABA_A_-mediated inhibitory postsynaptic currents (IPSCs) elicited by extracellular stimulation of inhibitory interneurons within the molecular layer were significantly altered in Ngsk *Prnp*^0/0^ PCs (Fig. 2). The rise time constant was significantly larger (i.e., IPSC rise was slower, *p* < 0.001) in Ngsk *Prnp*^0/0^ mice (3.09 ± 0.20 ms, n = 10) than in control mice (1.98 ± 0.19 ms, n = 10), while the decay-phase time constant did not differ significantly between the two groups (21.89 ± 2.32 ms, n = 10 for control slices vs 28.45 ± 2.34 ms, n = 10 for Ngsk *Prnp*^0/0^ slices, *p* = 0.062). Additionally, the averaged amplitude of IPSCs in Ngsk *Prnp*^0/0^ PCs was smaller than that in controls (80.1 ± 9.0 pA, n = 10 for control slices vs 44.1 ± 6.4 ms, n = 10 for Ngsk *Prnp*^0/0^ slices, *p* = 0.023) without a different in the paired-pulse ratio (PPR) (1.00 ± 0.07, n = 10 for control slices vs 1.15 ± 0.11, n = 10 for Ngsk *Prnp*^0/0^ slices, *p* = 0.74). Thus, GABA_A_-mediated IPSC was slower and weaker in Ngsk *Prnp*^0/0^ PCs.

### Reduced cerebellar LTD in Ngsk *Prnp*^0/0^ PCs

**Figure 3:**
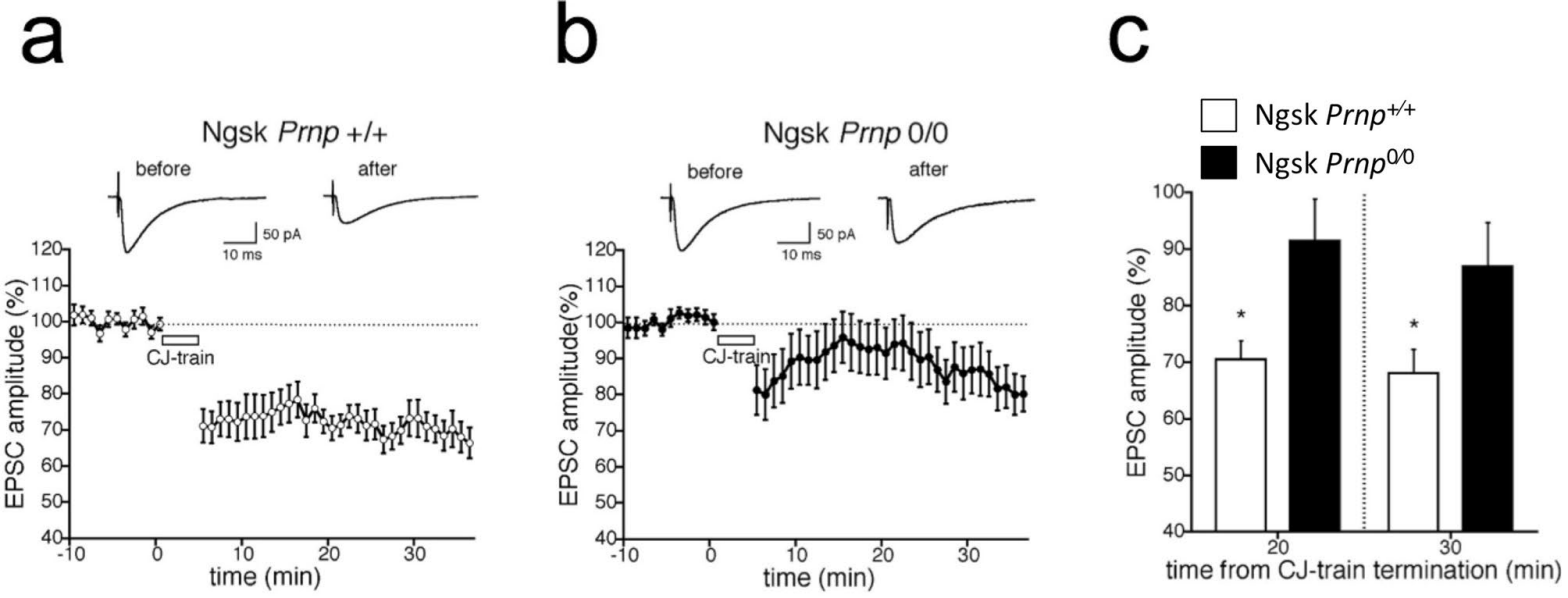
Cerebellar long-term depression is impaired in Ngsk *Prnp*^0/0^ mice. (**a**, **b**) Amplitude of PF-EPSCs in PCs from control (**a**) and Ngsk *Prnp*^0/0^ (**b**) mice. Cerebellar long-term depression (LTD) induced by conjunction protocol was inducible in control mice (open circle, *n* = 13 from 8 mice), but reduced in Ngsk *Prnp*^0/0^ mice (closed circle, *n* = 12 from 7 mice). The EPSC was evoked by stimulation of PF at 0.2 Hz throughout the experiments. Depolarization to + 20 mV for 200 ms was applied 300 times in conjunction with PF stimulation (CJ) over 5 min, as indicated by the empty bar. Insets are averages of individual EPSCs recorded before CJ stimulation and 25 min after CJ stimulation. (**c**) The amplitude of averaged EPSC at 20 or 30 min after CJ-train termination. Data points represent the mean ± SEM.

Next, to test the cerebellar LTD of synaptic transmission between PFs and PCs in Ngsk *Prnp*^0/0^ mice, we recorded PF-EPSCs from cerebellar PCs in the control and Ngsk *Prnp*^0/0^ mice and induced the LTD of PF-EPSCs by a conjunctive stimulation (CJ-train) protocol composed of 300 PF stimuli in conjunction with a depolarizing pulse to PCs (200 ms, – 60 to + 20 mV) repeated at 1 Hz^21–23^. In 12 of 13 PCs from control mice, CJ stimulation reduced the amplitude of PF-EPSCs; this depression (> 10% reduction) persisted for more than 30 min after the onset of stimulation (Fig. 3a). The mean percentage amplitude of PF-EPSCs measured at 25–30 min after CJ stimulation was 69.9 ± 4.3% of the original baseline (*n* = 13 from 8 mice, three cells studied blind). By contrast, PCs in Ngsk *Prnp*^0/0^ mice exhibited attenuated LTD of PF-EPSCs after CJ stimulation (Fig. 3b). Indeed, in 6 of 12 PCs from Ngsk *Prnp*^0/0^ mice, no significant depression of the EPSC was observed. The mean percentage amplitude of PF-EPSCs recorded at 25–30 min after CJ stimulation was 82.9 ± 6.9% (*n* = 12 from 7 mice, two cells studied blind). There was a significant difference in the magnitude of LTD between control and Ngsk *Prnp*^0/0^ mice (Mann–Whitney U test, *p* < 0.05), indicating that LTD-induction is impaired in Ngsk *Prnp*^0/0^ mice (Fig. 3c).

### Altered delay eyeblink conditioning in Ngsk *Prnp*^0/0^ mice at 7–8 weeks of age

**Figure 4:**
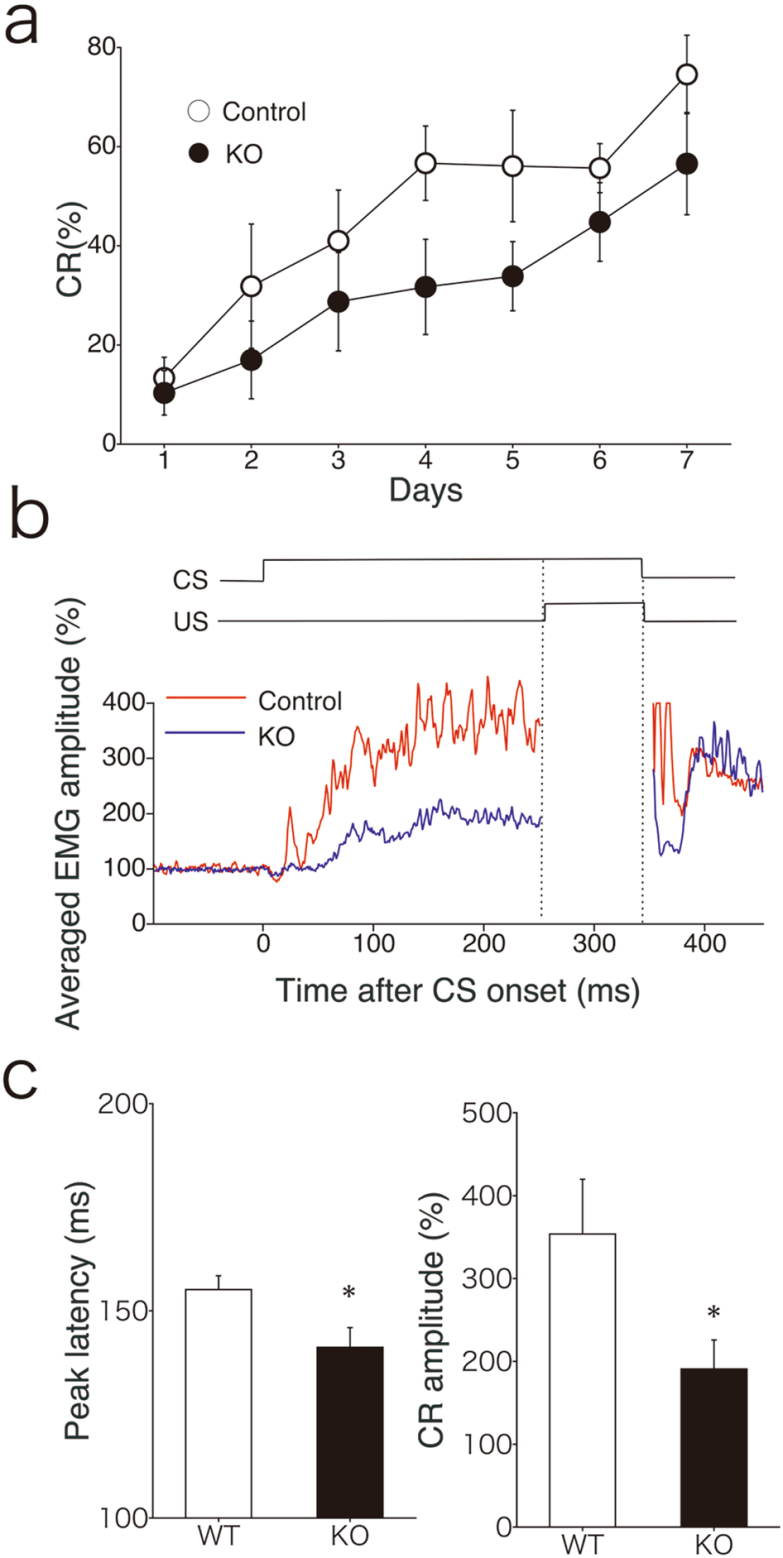
Altered delay eyeblink conditioning in young *Prnp*^0/0^ mice (7–8-week-old). (**a**) CR% for control (open circle, n = 8) and Ngsk *prnp*^0/0^ (closed circle, n = 8) mice. (**b**) Schematic representation of stimulus contingencies and timing for the delay eyeblink conditioning paradigm used in the present study. In the delay paradigm, the CS and US temporally overlap and terminate simultaneously. Averaged EMG amplitudes on days 7 were placed under the CS-US presentation. All EMG amplitudes obtained in one session (100 trials) were summed, representing the overall response pattern. (**c**) Averaged peak latency (left) and CR amplitude (right) for control (open bar, n = 8) and Ngsk *prnp*^0/0^ (closed bar, n = 8) mice. Results indicate the mean ± SEM. *p < 0.05.

Finally, we examined delay eyeblink conditioning, a form of cerebellum-dependent discrete motor learning, in Ngsk *Prnp*^0/0^ mice aged 7–8 weeks, which were almost the age of mice used for electrophysiological studies. With conditioning, the animals learn the adaptive timing of eye blinking; in our study, the conditioned response frequency (CR%) for the control mice progressively and significantly increased to over 70% on day 7 (Fig. 4a). On the contrary, CR% for Ngsk *Prnp*^0/0^ mice did not reach 60%. However, repeated measures ANOVA failed to reveal significant differences between the two groups (session × group interaction, F(6, 84) = 0.58, *p* = 0.57, a genotypic effect, F(1, 14) = 2.75, p = 0.12). The normalized electromyographic (EMG) amplitude on day 7 for Ngsk *Prnp*^0/0^ mice seemed to be lower than that for the control mice (Fig. 4b). Indeed, CR amplitude on day 7 was significantly reduced in Ngsk *Prnp*^0/0^ mice (*p* = 0.023, Fig. 4c, right panel). Peak latency was also significantly decreased in Ngsk *Prnp*^0/0^ mice compared to that in the control mice (*p* = 0.031, Fig. 4c, left panel). These results confirmed that the timing and amplitude of conditioned eyeblink response were altered in juvenile Ngsk *Prnp*^0/0^ mice that had not yet undergone PC degeneration.

## Discussion

Several *Prnp* null mutant mouse strains, including *Ngsk,* have been questioned in the field as to whether their phenotype is physiological^24^. Indeed, given that many different PrP knockout mouse strains produce diverse physiological phenotypes, most of the PrP knockout mouse strains have disparate findings because the *Prnp*^−/−^ locus is surrounded by genes other than Prnp itself ("flanking genes"), which it is thought to be indicative of^2,5,7^. Nevertheless, the present study aimed to specifically characterize the electrophysiological properties of Ngsk *Prnp*^0/0^ mice strain, which exhibits cerebellar PC degeneration and motor learning deficits.

The present electrophysiological study shows that cerebellar LTD is significantly impaired in Ngsk *Prnp*^0/0^ mice (Fig. 3). Furthermore, our behavioral study indicated that Ngsk *Prnp*^0/0^ mice (7–8-week-old) exhibited abnormalities in CR amplitude and CR timing, without a significant difference in CR probabilities (Fig. 4). These behavioral results replicate those obtained in experiments with Ngsk *Prnp*^0/0^ mice at 16 weeks old^10^; however, the extent of their learning disability appears to be more pronounced. The reason for the severe impairments of 7–8-week-old mice is unclear. In Ngsk *Prnp*^0/0^ mice, GFAP begins to increase gradually from 7 to 8 weeks of age^25^. GFAP is thought to be a significant factor needed for proper communication between Bergmann glia and PC, enabling occurrence of LTD^26^. Indeed, GFAP KO mice exhibited the impairments of cerebellar LTD and eyeblink conditioning^27^. Hence, one possibility is that in 16-week-old Ngsk mutants, improved LTD due to increased GFAP may have had a milder impact on the motor learning disabilities. Regardless, the present results are consistent with those of previous studies suggesting parallelism between impaired cerebellar LTD and altered delay eyeblink conditioning^28–32^, although the extent of impairment in both conditioned eyeblink response and cerebellar LTD appeared to be less than that observed in the previous reports^28–30,32^. As in the past, the results of the present study do not demonstrate a direct causal relationship between LTD and eyeblink conditioning, but suggest that there is a common molecular basis for both.

In addition, the effects of molecular layer interneuron-PC feed-forward inhibition (FFI) and absence of FFI on LTD formation have been studied in mice that are genetically deficient in inhibitory synaptic inputs to the PC^33^. The excitatory/inhibitory (E/I) ratio of PCs in these mice appears to be imbalanced, resulting in smaller action potential variability and loss of temporal fidelity of PC responses to parallel fiber stimulation. Furthermore, although LTD formation was normal in these mice, the vestibulo ocular reflex was impaired^33^. Eyeblink conditioning was also found to be impaired in the same mice^34^. Ngsk *Prnp*^0/0^ mice exhibited not only impaired LTD but also weakened GABA_A_ receptor- mediated inhibition in the cerebellum. Thus, alternatively, the dysfunction of inhibitory synaptic transmission in the molecular layer could be also responsible for the impairment of CR acquisition or timing^35^. IPSCs and long-term potentiation in hippocampal CA1 pyramidal cells of ZrchI *Prnp*^0/0^ mice have been reported to be abnormalities^14^, but the cerebellar LTD has not been clarified in the ZrchI *Prnp*^0/0^ mice. Previous studies suggested PrP-mediated several possible mechanisms underlying the regulation of cerebellar LTD and cellular toxicity^19,20,36–39^, e.g., PrP can bind to mGluR1 and modulate its function to prevent irregular Ca^2+^ signalling^39^. Because mGluR1 expressed in PCs is essential for both LTD induction and eyeblink conditioning^28,40,41^, the deficiency in PrP-mediated regulation of mGluR1 may be responsible for the impaired cerebellar plasticity in Ngsk *Prnp*^0/0^ mice.

**Figure 5:**
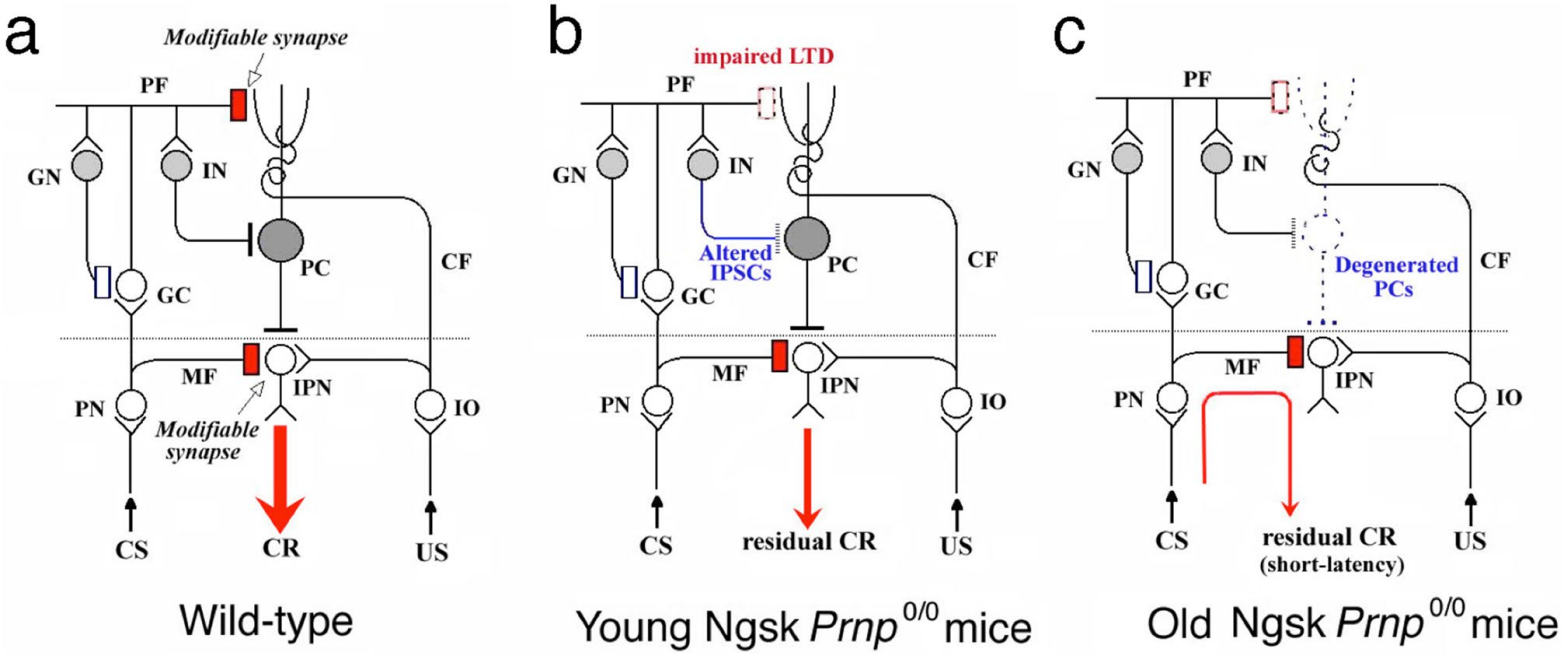
Schematic model for age-dependent impairment of cerebellar motor learning in Ngsk *Prnp*^0/0^ mice. The synaptic organization of the cerebellum underlying eyeblink conditioning is schematically shown. (**a**) In wild-type mice, synaptic plasticity in both PF-PC synapses and MF-IPN synapses (red oblong) contributes to the expression of CRs, whereas GN-GC synapse (blue oblong) is considered as the locus for regulating CR timing^47,48^. (**b**) In young Ngsk *Prnp*^0/0^ mice, cerebellar LTD between PF and PC was reduced and IN-PC synapse transmission was altered. (i) Weakened PF-LTD was correlated with impairment of CR acquisition and/or timing. (ii) Altered IPSCs induce excessive excitement of PCs. (**c**) In old Ngsk *Prnp*^0/0^ mice, PCs were degenerated by the excitotoxic mechanism due to weakened IPSCs. (i) Severe impairment of CR acquisition occurred because of degeneration of the cerebellar cortex. (ii) The short latency of residual CR is attributable to the plasticity at MF-IPN synapses in the absence of PCs^49^. *CF* climbing fibre, *GC* granule cell, *GN* Golgi neuron, *IO* inferior olive, *IN* inhibitory interneurons such as basket or stellate cells, *IPN* interpositus nuclei, *MF* mossy fibre, *PN* pontine nuclei. Open and closed circles represent excitatory and inhibitory neurons, respectively.

Histological changes in Ngsk *Prnp*^0/0^ mice occur at an age of approximately 40 weeks^42^, and a molecular mechanism underlying neuronal degeneration induced by ectopic expression of PrPLP/Dpl has not been identified, although some hypotheses have been suggested^43–46^. In the present study, we found that IPSCs in cerebellar PCs were altered in Ngsk *Prnp*^0/0^ mice, whereas a previous report showed different results using the ZrchI *Prnp*^0/0^ mouse cerebellum^15^. The discrepancies in IPSCs between the two types of the mutant mice can be explained by ectopic expression of PrPLP/Dpl in Ngsk *Prnp*^0/0^ mice. Our result is rather similar to that the ZrchI *Prnp*^0/0^ mouse hippocampus exhibits a reduction in GABA_A_ receptor-mediated fast inhibition, suggesting that PrP^C^ plays a key role in normal inhibitory postsynaptic function^14^ and implies that excessive excitement of PCs induced by suppressing inhibitory inputs via ectopic overexpression of PrPLP/Dpl inhibits the maintenance of PCs in these Ngsk *Prnp*^0/0^ mice. Thus, impairment of CR acquisition in old Ngsk *Prnp*^0/0^ mice could be caused by a secondary effect of PrPLP/Dpl overexpression, particularly the loss of cerebellar PCs. Figure 5 illustrates such a schematic model for age-dependent alterations of delay eyeblink conditioning in Ngsk *Prnp*^0/0^ mice, from the present study and previous reports^10^.

There is at least one other possible explanation for alteration of eyeblink conditioning in young Ngsk *Prnp*^0/0^ mice. Since normal prion protein is expressed on all neuronal cell types in the cerebellum^11^, alterations in GABA_A_ receptor-mediated IPSCs could be true in other types of cerebellar neurons other than Purkinje cells. Thus, GABAergic transmission at Golgi-granule cell synapses may be modified in PrP^C^-null mice. Considering the timing model described in previous studies^47,50,51^, functional alterations in synaptic transmission between Golgi cells and granule cells may be a factor causing changes in learning-dependent CR timing in young Ngsk and ZrchI *Prnp*^0/0^ mice. Their altered CR timing may be attributed to abnormal regulation of the granule cells by Golgi cells (Fig. 5b). This explanation is consistent with a previous immunohistochemical study indicating that PrP^C^ is most highly expressed in the axon terminals of granule cells in the cerebellum^52^. Therefore, the physiological properties of granule cells in Ngsk *Prnp*^0/0^ mice should be further examined. Furthermore, because aberrant timing of eyeblink conditioning was also observed in *Zrch* I mice^10^, it is important to investigate the physiology of granule cells in Zrch III mice on a pure C57BL/6J genetic background for future studies^53^.

An appropriate E/I balance is essential for normal adult brain function^54,55^. Thus E/I imbalance affects the normal function and disrupts synchronization between various circuit elements, and can cause autism spectrum disorder (ASD)^56,57^, schizophrenia^58,59^, and Alzheimer's disease (AD)^60^. Indeed, several studies on their model mice have elaborated on the correlation between decreased IPSCs and neuronal death^61,62^. In most human prion diseases, including Creutzfeldt-Jakob disease (CJD), neuronal loss in the cerebellum and abnormal PrP deposition are major neuropathological findings. Furthermore, epileptic-like symptoms or abnormal waves in EEG are often observed in patients with CJD^63–65^. Therefore, the Ngsk *Prnp*^0/0^ mouse model may help in studying the mechanisms underlying synaptic loss and neurodegeneration in the cerebellum resulting from the loss of PrP^c^ and ectopic expression of PrPLP/Dpl^66^.

## Materials and methods

### Subjects

Ngsk *Prnp*^0/0^ mice were obtained as described previously^36^. Male F3 Ngsk *Prnp*^0/0^ mice were crossed with female C57BL/6J mice (purchased from CLEA Japan, Tokyo, Japan), producing F4 heterozygous mice (*Prnp*^+/0^ mice). The mutant mice (Ngsk *Prnp*^0/0^) and their littermate controls (Ngsk *Prnp*^+/+^) were derived by inter-crossing F4 Ngsk *Prnp*^+/0^ male and female mice. Their genotypes were confirmed by polymerase chain reaction (PCR) amplification of genomic DNA extracted from the tail of each mouse using specific primers for the mouse PrP gene in a 346-base pair PCR fragment (5′-CCGCTACCCTAACCAAGTGT-3′ and 5′-CCTAGACCACGAGAATGCGA-3′) and neomycin-resistant gene (5′-GGTGCCCTGAATGAACTGCA-3′ and 5′-GGTAGCCGGATCAAGCGTAT-3′), resulting in a 227-base pair PCR fragment). All subjects were maintained on a 12-h:12-h dark: light cycle with food and water available ad libitum. All animal procedures were performed in accordance with the guidelines for animal experimentation from the ethical committee of The University of Tokyo and Tokushima Bunri University. The experimental protocol was approved by the guidelines for the care and use of experimental animals in the animal investigation committee at Tokushima Bunri University, and the animal welfare committees of The University of Tokyo. In addition, the minimum number of required animals was used for these experiments, and efforts were made to minimize pain.

### Electrophysiology

Cerebellar slices from 3–6-week-old Ngsk *Prnp*^+/+^ (control) and Ngsk *Prnp*^0/0^ mice were prepared as described previously^67^. The mice were treated with CO_2_ and decapitated. Sagittal slices (180–200-μm thick) of the cerebellar vermis were prepared with a microslicer (DTK-1000, Dosaka, Japan) in ice-cold extracellular solution containing (in mM) 252 sucrose, 3.35 KCl, 21 NaHCO_3_, 0.6 NaH_2_PO_4_, 9.9 glucose, 1 CaCl_2_, and 3 MgCl_2_ and gassed with a mixture of 95% O_2_ and 5% CO_2_ (pH 7.4). The slices were maintained at room temperature in artificial cerebrospinal fluid (ACSF) containing (in mM) 125 NaCl, 2.5 KCl, 26 NaHCO_3_, 1.25 NaH_2_PO_4_, 20 glucose, 2 CaCl_2_, and 1 MgCl_2_ (bubbled with a mixture of 95% O_2_ and 5% CO_2_ to maintain the pH at 7.4). Whole-cell voltage-clamp recordings were made from visually identified PCs under Nomarski optics using a water-immersion objective lens (40 ×, NA 0.75, Zeiss, Oberkochen, Germany). Patch pipettes (3–4 MΩ) were filled with intracellular solution containing (in mM) 150 KCH_3_SO_3_, 5 KCl, 0.3 K-EGTA, 5.0 Na-HEPES, 3.0 Mg-ATP, and 0.4 Na-GTP (pH 7.4). Membrane currents were recorded using an EPC-7 amplifier (List Electronic, Darmstadt, Germany) and pCLAMP software (Molecular Devices, Sunnyvale, CA, USA), and then digitized and stored on a computer disk for off-line analysis. All signals were filtered at 2–4 kHz and sampled at 5–20 kHz. PF-mediated ionotropic glutamate receptor-type EPSCs were identified based on their response properties following paired-pulse stimulation (duration, 0.05–0.1 ms; amplitude, 5–15 V) applied via a glass microelectrode with a 2–3-μm tip diameter filled with normal ACSF placed within the molecular layer in the cerebellar cortex. Paired-pulse stimulation was applied at 0.2 Hz. To measure PF-evoked EPSCs, bicuculline (10 μM) was added to the ACSF to eliminate IPSCs. Series resistance (8–18 MΩ) was compensated by 60–70% and monitored using a 5-mV hyperpolarizing voltage step after PF stimulation. Cerebellar LTD was induced following a conjunctive stimulation (CJ-train) protocol composed of 300 PF stimuli in conjunction with a depolarizing pulse (200 ms, from – 60 to + 20 mV) repeated at 1 Hz. To measure monosynaptic GABA_A_-mediated IPSCs, 6-cyano-7-nitroquinoxaline-2,3-dione (10 μM), D(-)-2-amino-5-phosphonopentanoic acid (100 μM), and CGP35348 (500 µM) were added to block excitatory synaptic transmission and GABA_B_ receptor responses. The membrane of the PCs was held at –50 mV, and IPSCs were evoked by paired-pulse stimulation (duration, 0.1 ms; amplitude, 10 V) with a glass microelectrode (tip diameter, 2 μm) filled with ACSF and placed within the molecular layer. All physiological experiments were performed at room temperature (24–26 °C).

### Eyeblink conditioning

For the behavioural study, 7–8-week-old Ngsk *Prnp*^+/+^ (control) and Ngsk *Prnp*^0/0^ male mice were used. The surgery was performed as described previously^32,68,69^. The mice were anesthetized with ketamine (80 mg/kg, i.p. Sankyo, Tokyo, Japan) and xylazine (20 mg/kg, i.p. Bayer, Tokyo, Japan). Four Teflon-coated stainless-steel wires (100 µm in diameter, A-M Systems, WA, USA) were implanted subcutaneously under the left eyelid. Two of the wires were used to deliver the US and the remaining two to record an electromyogram (EMG) from the musculus orbicularis oculi, which is responsible for eyelid closure. Here, we modified the conventional EMG procedure to improve the sensitivity for detecting MOO activities. The mice were trained in delay eyeblink conditioning, in which the CS overlaps and coterminates with the US, for seven days. A tone of 352 ms duration (1 kHz, 80 dB) was used as CS and electrical shock with 100 ms duration (100 Hz square pulses) as US. The US intensity was carefully determined, and the minimal current amplitude required to elicit an eyeblink response with constant amplitude was adjusted daily for each animal (less than 0.5 mA). Experiments were conducted during the light phase of the LD cycle in a container (10 cm in diameter) placed in a sound- and light-attenuating chamber. Daily acquisition training consisted of 100 trials grouped in 10 blocks. Conditioning sessions consisted of 10 CS-only (every 10th trial) and 90 CS-US paired trials. The CR amplitude was calculated as the average amplitude over the 50 ms period just before the US. Data were analyzed as described previously^10,30^.

### Statistical analysis

All data and samples were analyzed by an individual blinded to the genotype. Unpaired t-tests or the Mann–Whitney test were used. Data for eyeblink conditioning were analyzed by the two-way repeated measures ANOVA to assess the effects of genotype and/or session. The difference was considered significant when the *P* value was less than 0.05. Tests were performed using Excel or GraphPad Prism 6 (GraphPad Software, Inc., La Jolla, CA). All data are displayed as mean ± standard error of the mean (SEM).
